# Specifications of standards in systems and synthetic biology: status and developments in 2020

**DOI:** 10.1515/jib-2020-0022

**Published:** 2020-06-29

**Authors:** Falk Schreiber, Björn Sommer, Tobias Czauderna, Martin Golebiewski, Thomas E. Gorochowski, Michael Hucka, Sarah M. Keating, Matthias König, Chris Myers, David Nickerson, Dagmar Waltemath

**Affiliations:** Dept. of Computer and Information Science, University of Konstanz, Konstanz, Germany; Faculty of Information Technology, Monash University, Melbourne, Australia; Royal College of Arts, London, UK; Heidelberg Institute for Theoretical Studies (HITS), Heidelberg, Germany; School of Biological Sciences, University of Bristol, Bristol, UK; California Institute of Technology, Pasadena, USA; EBI Hinxton, Cambridge, UK; Institute for Theoretical Biology, Humboldt-University Berlin, Berlin, Germany; Dept. of Electrical and Computer Engineering, University of Utah, Salt Lake City, USA; Auckland Bioengineering Institute, University of Auckland, Auckland, New Zealand; University Medicine Greifswald, Greifswald, Germany

**Keywords:** ontologies, standards, systems biology, synthetic biology

## Abstract

This special issue of the *Journal of Integrative Bioinformatics* presents papers related to the 10th COMBINE meeting together with the annual update of COMBINE standards in systems and synthetic biology.

## 1 Introduction

COMBINE (‘COmputational Modelling in BIology’ NEtwork) [1], [2], the formal entity which coordinates the development of standards in systems and synthetic biology, celebrated 10 years of activity in 2019. COMBINE not only coordinates standard developments, but also fosters and moderates discussions; designs and implements dissemination strategies; and organises two annual community meetings – COMBINE and HARMONY.

This special issue contains two papers, one new standard, and six updates of standards. Waltemath et al. [3] discuss the first 10 years of the international coordination network for standards in systems and synthetic biology, and summarises the COMBINE meeting in Heidelberg in July 2019. Brunak et al. [4] present ongoing works and open questions towards standardisation guidelines for *in silico* approaches in personalised medicine.

**Figure 1:**
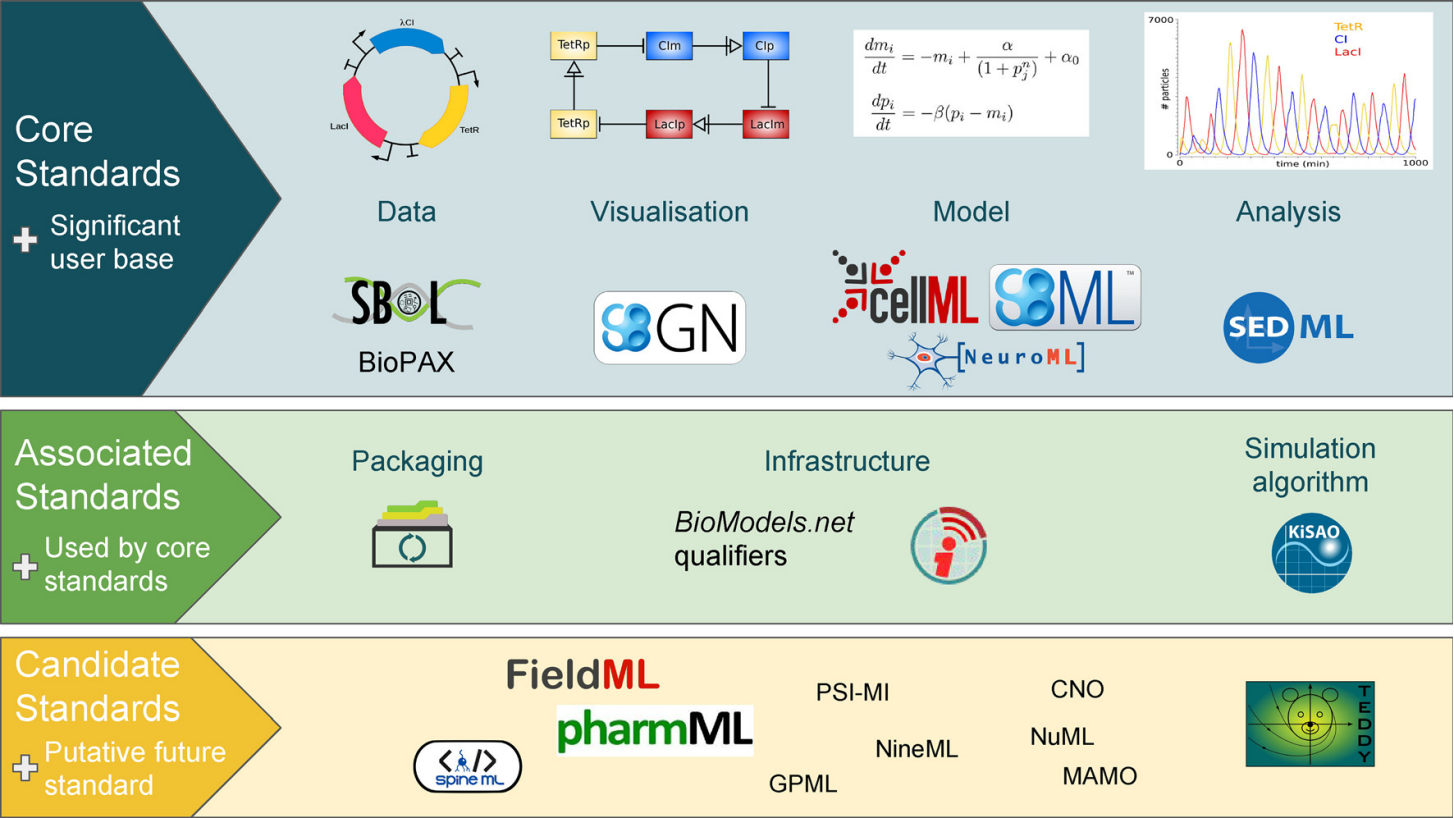
Standards and associated efforts in systems and synthetic biology (from Ref. [7]).

COMBINE standards and associated initiatives cover a wide range of disciplines, see Figure 1. This special issue only highlights updates over the last year, namely the CellML 2.0 specification, the Systems Biology Graphical Notation - Markup Language (SBGN-ML) Milestone 3, the SBML (Systems Biology Markup Language) Level 3 Packages “Distrib” and “Multi”, the SBML (Systems Biology Markup Language) Version 3.0.0, and the SBOL (Synthetic Biology Open Language) Visual Version 2.2. Additionally, one new standard has officially been added, Open Modelling EXchange format (OMEX) metadata specification 1.0, to harmonise the descriptions of metadata.

Further information on all standards and activities as well as links to the community websites are available from the COMBINE web site at https://co.mbine.org/. Detailed overviews of COMBINE, its history and its organisation have been provided, for example, by Hucka et al. [1], Myers et al. [2] or Waltemath et al. [5] . The annual special issue on COMBINE standards has become a tradition since its launch in 2016. Earlier editions provide summaries of updates for the years 2015–18 [6], [7], [8], [9].

We hope that this editorial is helpful in identifying the relevant specification documents for standards in systems in synthetic biology in the year 2020.

## 2 Current versions of COMBINE standards

Please refer to the following specifications when using COMBINE standards. New specifications or updates of existing specifications are highlighted with *NEW*.

### 2.1 Core standards

#### 2.1.1 BioPAX (Biological PAthway eXchange)

Biological PAthway eXchange (BioPAX) is a standard language for integration, exchange and analysis of biological pathway data. It is expressed in OWL. The current specification is listed in Table 1.

**Table 1:** Latest specifications for BioPAX.

Standard	Specification	Reference
BioPAX [10]	BioPAX	[11]

#### 2.1.2 CellML

The CellML language is an XML markup language to store and exchange computer-based mathematical models. The current specifications are listed in Table 2.

**Table 2:** Latest specifications for CellML.

Standard	Specification	Reference
CellML [13]	CellML 2.0	[12]
	CellML Metadata Framework 2.0	[14]
	CellML 1.1	[15]

*NEW* CellML 2.0 [12]: The development of CellML 2.0 was guided by observing the use of CellML 1.1 in the community for over 10 years. The syntax of CellML 1.1 has been clarified in areas where discrepancies in model interpretation are often seen and simplified to remove features that are never used. These enhancements are primarily aimed at improving the model reuse capabilities of CellML. The single substantial addition to CellML that is introduced in CellML 2.0 is the concept of *resets*, rules that define a change in model state dependent on specified conditions being met during a simulation experiment.

#### 2.1.3 NeuroML (Neural Open Markup Language)

The NeuroML is an XML-based description language that provides a common data format for defining and exchanging descriptions of neuronal cell and network models. The current specification is listed in Table 3.

**Table 3:** Latest specifications for BioPAX.

Standard	Specification	Reference
NeuroML [16], [17]	NeuroML version 2.0	[16]

#### 2.1.4 SBGN (Systems Biology Graphical Notation)

The SBGN, is a set standard graphical languages to describe visually biological knowledge. It is currently made up of three languages describing Process Descriptions, Entity Relationships and Activity Flows. In addition, SBGN-ML is an XML-based file format describing the geometry of SBGN maps, while preserving their underlying biological meaning. The current specifications are listed in Table 4.

**Table 4:** Latest specifications for SBGN.

Standard	Specification	Reference
SBGN [19]	SBGN Process Description Level 1 Version 2	[20]
	SBGN Entity Relationship Level 1 Version 2.0	[21]
	SBGN Activity Flow Level 1 Version 1.2	[22]
	SBGN Markup Language Version 0.3	[18]

*NEW* SBGN-ML Milestone 3 [18] includes new developments, such as support for multiple SBGN maps within a single file, complete support for the submap glyph, and the possibility to store colours and annotations through extensions. In addition, the *language* attribute has been deprecated to add a more detailed *version* attribute and the SBGN AF perturbation glyph has been deprecated to align with the SBGN AF specification.

#### 2.1.5 SBML (Systems Biology Markup Language)

The SBML is a computer-readable XML format for representing models of biological processes. SBML is suitable for, but not limited to, models using a process description approach. SBML development is coordinated by an elected editorial board and central developer team. The current specifications are listed in Table 5.

**Table 5:** Latest specifications for SBGN.

Standard	Specification	Reference
SBML [23]	SBML Level 3 Core, Version 2 Release 2	[24]
	SBML Level 3 Package: Distributions, Version 1, Release 1	[25]
	SBML Level 3 Package: Flux Balance Constraints, Version 2	[26]
	SBML Level 3 Package: Groups, Version 1	[27]
	SBML Level 3 Package: Hierarchical Model Composition, Version 1	[28]
	SBML Level 3 Package: Layout, Version 1	[29]
	SBML Level 3 Package: Multistate, Multicomponent and Multicompartment Species, Version 1, Release 2	[30]
	SBML Level 3 Package: Qualitative Models, Version 1	[31]
	SBML Level 3 Package: Render, Version 1, Release 1	[32]

*NEW* SBML Level 3 Package: Distributions, Version 1, Release 1 [25] introduces distributions and uncertainties to SBML. Biological models often contain elements that have inexact numerical values, since they are based on values that are stochastic in nature or data that contains uncertainty. The SBML Level 3 Core specification does not include an explicit mechanism to include inexact or stochastic values in a model, but it does provide a mechanism for SBML packages to extend the Core specification and add additional syntactic constructs. The SBML Distributions package for SBML Level 3 adds the necessary features to allow models to encode information about the distribution and uncertainty of values underlying a quantity.

*NEW* SBML Level 3 Package: Multistate, Multicomponent and Multicompartment Species, Version 1 Release 2 [30] addresses some issues raised by users about unclear aspects of Release 1 of the specification; it also clarifies the use of XML namespaces, and updates example models.

#### 2.1.6 SBOL (Synthetic Biology Open Language)

The SBOL is a language for the description and the exchange of synthetic biological parts, devices and systems. The current specifications are listed in Table 6.

**Table 6:** Latest specifications for SBOL.

Standard	Specification	Reference
SBOL [35]	SBOL Version 3.0.0	[33]
	SBOL Visual Version 2.2	[34]

*NEW* SBOL Version 3.0.0 [33] condenses and simplifies previous versions of SBOL based on experiences in deployment across a variety of scientific and industrial settings. In particular, SBOL 3.0.0, (1) separates sequence features from part/sub-part relationships, (2) renames ComponentDefinition/Component to Component/SubComponent, (3) merges Component and Module classes, (4) ensuring consistency between data model and ontology terms, (5) extends the means to define and reference SubComponents, (6) refines requirements on object URIs, (7) enables graph-based serialisation, (8) moves Systems Biology Ontology (SBO) for Component types, (9) makes all sequence associations explicit, (10) makes interfaces explicit, (11) generalises SequenceConstraints into a general structural Constraint class, and (12) expands the set of allowed constraints.

*NEW* Synthetic Biology Open Language Visual (SBOL Visual) Version 2.2 [34] is a refinement to the standard harmonising the ontology used and extending the glyph library to capture new biological parts. Specifically, the changes in SBOL Visual 2.2. include, (1) the grounding of the molecular species glyphs is changed from BioPAX to SBO to better align with the use of SBO terms for interaction glyphs, (2) new glyphs are added for proteins, introns, and polypeptide regions (e. g. protein domains), (3) the prior recommended macromolecule glyph is deprecated in favour of its alternative, and (4) small polygons are proposed as alternative glyphs for simple chemicals.

#### 2.1.7 SED-ML (Simulation Experiment Description Markup Language)

The Simulation Experiment Description Markup Language (SED-ML) is an XML-based format for encoding simulation experiments. SED-ML allows to define the models to use, the experimental tasks to run and which results to produce. SED-ML can be used with models encoded in several languages, as long as they are in XML. The current specification is listed in Table 7.

**Table 7:** Latest specifications for SED-ML.

Standard	Specification	Reference
SED-ML [36]	SED-ML Level 1 Version 3	[37]

### 2.2 Associated standards

Associated standards provide an additional layer of semantics to COMBINE representation formats. The current specifications are listed in Table 8.

**Table 8:** Latest specifications of associated standards.

Associated standard	Specification	Reference
COMBINE Archive [39]	COMBINE Archive 1.0	[40]
OMEX Metadata	OMEX Metadata Version 1.0	[38]
BioModels.net qualifiers [41]		[42]
Identifiers.org URIs [43]		[44]
Systems Biology Ontology [45]	[external] Bioportal	[46]
Kinetic Simulation Algorithm	[external] Bioportal	[47]
Ontology [45]		

A COMBINE archive is a single file bundling the various documents necessary for a modelling and simulation project, and all relevant information. The archive is encoded using the OMEX. COMBINE archive metadata provides a harmonised, community-driven approach for annotating a variety of standardised model and data representation formats within a COMBINE archive. BioModels.net qualifiers are standardised relationships (predicates) that specify the relation between an object represented in a description language and the external resource used to annotate it. The relationship is rarely one-to-one, and the information content of an annotation is greatly increased if one knows what it represents, rather than only know it is “related to” the model component. MIRIAM Unique Resource Identifiers allow one to uniquely and unambiguously identify an entity in a stable and perennial manner. MIRIAM Registry is a set of services and resources that provide support for generating, interpreting and resolving MIRIAM URIs. Through the Identifiers.org technology, MIRIAM URIs can be dereferenced in a flexible and robust way.

MIRIAM URIs are used by SBML, SED-ML, CellML and BioPAX controlled annotation schemes. The SBO is a set of controlled, relational vocabularies of terms commonly used in Systems Biology, and in particular in computational modelling.

Each element of an SBML file carries an optional attribute sboterm which value must be a term from SBO. Each symbol of SBGN is associated with an SBO term.

The Kinetic Simulation Algorithm Ontology (KiSAO) describes existing algorithms and their inter-relationships through their characteristics and parameters.

Kinetic Simulation Algorithm Ontology is used in SED-ML, which allows simulation software to automatically choose the best algorithm available to perform a simulation and unambiguously refer to it.

*NEW* The OMEX Metadata Specification Version 1.0 [38] has been developed to harmonise the way in which computational models and other types of modelling files are annotated. Guided by consensus across the COMBINE community, the specification provides technical guidelines for encoding metadata that describes the contents of modelling projects within OMEX-formatted COMBINE archives. By helping to ensure that computable knowledge about modelling projects is encoded and shared in a consistent manner across the broader modelling community, the specification can help promote model reuse, reproducibility, discovery and semantics-based analyses.
